# The occurrence rate of acute-onset postoperative endophthalmitis after cataract surgery in Chinese small- and medium-scale departments of ophthalmology

**DOI:** 10.1038/srep40776

**Published:** 2017-01-17

**Authors:** Yanan Zhu, Xinyi Chen, Peiqing Chen, Jianjun Wu, Huixia Hua, Ke Yao

**Affiliations:** 1 Eye center, 2nd Affiliated Hospital of Medical College, Zhejiang University, Zhejiang, China; 2 Department of Mathematics and Statistics, College of Science, Zhejiang University, Zhejiang, China

## Abstract

Endophthalmitis can be a devastating complication after cataract surgery. Therefore, this study sought to better understand the occurrence rate of acute-onset postoperative endophthalmitis after cataract surgery in Chinese small and medium-scale departments of ophthalmology, as well as identify its risk factors and assess the treatment options. This investigation revealed 52 postoperative endophthalmitis cases in 46,185 operations at 30 hospitals from 2011 to 2013, at an occurrence rate of 0.11%. A small cataract surgery volume of less than 500 cases per year (OR 2.21; p = 0.006), the absence of 0.5% povidone iodine (PVP-I) irrigation (OR 1.73; p = 0.046), and intraoperative posterior capsular rupture (PCR) with vitreous loss (OR 4.40; p = 0.034) showed statistically significant associations with endophthalmitis in the multivariate analysis. The rate of culture positivity was 44.2%, with *Staphylococcus epidermidis* being the most common organism isolated in China. More than 40% of the endophthalmitis cases were treated with a nonstandard antibiotics regimen, and only 32.7% of these had a visual acuity of better than 20/40. We concluded that the occurrence rate of acute-onset endophthalmitis following cataract surgery in Chinese small and medium-scale departments of ophthalmology lags behind the level of developed countries, as well as Chinese top eye centers. Overall, the use of 0.5% PVP-I irrigation seemed to be an effective measure to reduce the risk of the development of postoperative endophthalmitis.

Currently, there are eleven million cataract patients in China, and this rate increases by 400,000 cases annually [statistics reported by the Ministry of Health of the People’s Republic of China (http://www.nhfpc.gov.cn)]. Last year, there were 1.9 million cataract surgeries performed^1^. Unfortunately, due to both social and economic factors, there are still large gaps in the levels of medical care provided among the different districts and hospitals in China. In a previous study published in the British Journal of Ophthalmology on July 24, 2013, we focused on top eye centers (cataract surgery volume ≥150 cases per month), which represent the most advanced medical care environment in China. We investigated the occurrence rate of acute-onset endophthalmitis after 201,757 cataract surgeries in eight centers between 2006 and 2011 (0.033%), which was close to that reported in developed countries^2^. Obviously, that study could not determine the overall status of the postoperative endophthalmitis incidence and treatment throughout the whole country. In order to reveal the status of most hospitals in China, the Board of the Chinese Cataract Society sampled 30 hospitals with small and medium-scale departments of ophthalmology, whose cataract surgery volume ranged from 10 to 100 cases per month. This was to investigate the occurrence rate of acute-onset endophthalmitis after cataract surgery, and assess the treatment standards. In addition, the study focused on effective infection prophylaxes and the associated risk factors.

## Results

From January 2011 to December 2013, 59,392 cataract surgeries were performed in these 30 departments. The average cataract surgery volume was 659.9 cases per year (range 175–1,199), and the overall follow-up rate of the patients at 2 months was 77.8%. Considering that the acute-onset postoperative endophthalmitis was diagnosed within 6 weeks, those patients without 2-month follow-up records were excluded. In total, 46,185 cataract surgeries were included in the analysis: 40,848 phacoemulsifications and 5,337 ECCEs. Fifty-two eyes of 52 patients developed acute-onset endophthalmitis following cataract surgery in these 46,185 operations, which was an occurrence rate of 0.11% (95% CI 0.08 to 0.14), and there were no significant differences among the three separate years. The 30 departments achieved different occurrence rates for postoperative endophthalmitis, ranging from 0.03% to 0.60% (Table 1).

Regarding the infection prophylaxes, 43.3% of the centers (13/30) trimmed the patients’ lashes, and 56.7% (17/30) irrigated with 0.5% PVP-I solution for the conjunctiva preparation. Antibiotics were added to the irrigation solution (16 mg/l tobramycin or 8 mg/l gentamicin) by 10.0% of the centers (3/30), while 86.7% of the centers (26/30) used tobramycin (subconjunctival injection or ointment) upon completing the surgery.

The mean age of these 52 patients (20 males, 32 females) was 68.3 years old (range 28–92). Among these 52 cases, five underwent ECCE and 47 underwent phacoemulsification. Seventeen of the patients were from hospitals whose cataract surgery volume was less than 500 cases per year. The occurrence of intraoperative posterior capsule rupture with vitreous loss was noted in three of the 52 patients. Twenty-five patients were treated without 0.5% PVP-I irrigation for the conjunctiva preparation, and the median interval between the surgery and diagnosis was 6.18 days (range 1–20). After removing two cases that lacked data regarding the patient’s presenting visual acuity, 92% of the cases (46/50) had visual acuities worse than 20/70 at presentation, while 74% (37/50) were worse than 20/1,000. Based upon the sampling results of the aqueous humor or vitreous body, the rate of culture positivity was 44.2% (23/52). Of the 20 culture-positive cases, *Staphylococcus epidermidis* (43.5%) was the most commonly isolated organism (Table 2). For the therapy, 59.6% of the cases (31/52) were treated with the proper antibiotics (according to the culture results, or with the use of broad-spectrum antibiotics), while 38.5% of the cases (20/52) received pars plana vitrectomy treatment. In addition, 51.9% of the cases (27/52) reported a final corrected distance visual acuity (CDVA) of 20/70 or better, but only 32.7% of these had a visual acuity of better than 20/40.

The multivariate analysis showed that an intraoperative posterior capsule rupture (PCR) with vitreous loss (OR = 4.40; 95% CI 1.34–14.14; p = 0.034), a small cataract surgery volume (less than 500 cases per year) (OR = 2.21; 95% CI 1.24–3.94; p = 0.006), and the absence of 0.5% PVP-I solution irrigation (OR = 1.73; 95% CI 1.01–2.98; p = 0.046) were independent risk factors for the development of postoperative endophthalmitis. To identify the relationship between PCR and postoperative endophthalmitis, we first analyzed this complication as a factor, but found there was no association between them (p = 0.614). Then, we divided this complication into two case types: PCR with vitreous loss and PCR without vitreous loss. We found that only the PCR with vitreous loss was associated with endophthalmitis (p = 0.034), while the PCR without vitreous loss was not a risk factor (p = 0.55). The age, gender, systemic disease, place of surgery, type of surgery, antibiotic irrigation, hospital geographic pattern, and general level of the location’s city were unrelated to the risk of endophthalmitis (Table 3).

Finally, we constructed a logistic regression model of the visual outcome (final BCVA of 20/70 or better) that included 9 predictors: age, gender, systemic disease, type of surgery, time interval to onset, presenting visual acuity, cultured results, pars plana vitrectomy, and antibiotic therapy. A presenting visual acuity of 20/1,000 or better (OR = 7.07; 95% CI 1.15–43.41; p = 0.035), proper antibiotic therapy (OR = 7.44; 95% CI 1.63–33.91; p = 0.01), and phacoemulsification (OR = 16.95; 95% CI 1.04–276.83; p = 0.047) were found to be significantly associated with a good visual outcome (final BCVA of 20/70 or better) in this multivariate analysis (Table 4).

## Discussion

These analyses determined the occurrence rate of acute-onset endophthalmitis secondary to cataract surgery in Chinese small and medium-scale departments of ophthalmology from 2011 to 2013. The occurrence rate of postoperative endophthalmitis was calculated to be 0.11% in this analysis of 46,185 cataract surgeries. When compared with our previous survey of Chinese top eye centers, which showed an occurrence rate of 0.033%, the occurrence rate found in this survey was over twice as high. Since the top centers only account for 3.1% of the hospitals in China [statistics reported by the National Bureau of statistics of China (http://data.stats.gov.cn)], the results of the current survey are more representative of the present medical treatment level in most Chinese hospitals. In developed countries, the incidence of postoperative endophthalmitis ranges from 0.012 to 0.076%^3^,^4^,^5^,^6^,^7^,^8^,^9^, which is far less than in our study. Recently, the European Society of Cataract & Refractive Surgeons (ESCRS) recommended the intracameral injection of 1 mg of cefuroxime at the close of cataract surgery^10^. In addition, one Spanish study showed a subsequent drop in endophthalmitis rates of approximately 15-fold (0.59% to 0.039%) after using intracameral cefuroxime (1 mg)^11^. A recent U.S. study also stated a dramatic reduction in infection rates (0.31% to 0.014%) after intracameral antibiotics became a standard prophylactic intervention (moxifloxacin or vancomycin was used in those cases of allergy)^12^. These findings lend tremendous support to the clinical benefits of intracameral antibiotic injections.

In China, intracameral antibiotic injections are not widely promoted, but this procedure is worth promoting to prevent postoperative endophthalmitis in Chinese small and medium-scale departments of ophthalmology. However, Witkin^13^ recently reported 11 cases of postoperative hemorrhagic occlusive retinal vasculitis occurring after cataract surgery. These cases were suspected to be caused by a delayed immune reaction after the use of prophylactic intracameral vancomycin. Considering Witkin’s concerns, vancomycin is not recommended in routine prophylactic intracameral injections during cataract surgery.

In U.S. and European studies, a high concentration (5% or 10%) of PVP-I was suggested to effectively lower the incidence of postoperative endophthalmitis^5^,^14^,^15^,^16^. However, in a Japanese study, 0.1–1% PVP-I was found to be more rapidly bactericidal than a full-strength solution (10%)^17^. Considering the toxicity of PVP-I to the corneal epithelium and endothelium^18^,^19^, the Chinese Cataract Society has recommended a low concentration application of PVP-I in the conjunctiva. Here, our results support the suggestion that 0.5% PVP-I could reduce the incidence of postoperative endophthalmitis in a large sample. Due to the increased levels of free iodine in a low concentration solution^18^, irrigation with a 0.5% PVP-I solution in the conjunctiva was beneficial for endophthalmitis prophylaxis. Moreover, a low concentration of PVP-I was found to be effective and safe for the preparation of the conjunctival sac^20^,^21^. It seems that a low concentration PVP-I irrigation is a simple and low-cost measure which is worth promoting; however, further randomized controlled trials need to be performed to evaluate the optimum concentration.

A hospital volume of less than 500 cataract surgeries per year was a significant risk factor that we identified for postoperative endophthalmitis (OR = 2.21). At some point, the small-scale departments may have had outdated equipment or surgery rooms. In other words, it is more important to promote the prophylaxis standards in small-scale departments of ophthalmology.

Previous research has shown an increased incidence of endophthalmitis following cataract surgery with clear corneal incisions, when compared with scleral tunnel incisions, due to the instability of the surgical wound^22^. Confusingly, recent studies have proposed that the incidence of postoperative endophthalmitis was not related to the incision location^7^,^23^. Here, in our study, we observed no relationship between the type of incision and the development of endophthalmitis (p = 0.718). We suggest that a watertight incision might be more important than the type of the incision.

This study has also put the medical levels in China into focus. We found that the ECCE surgery rate was much higher in the small and medium-scale departments when compared with the top eye centers in China (10.3% vs 2.2%)^2^. The low coverage of phacoemulsification suggests an imbalance of medical resources in China, including the lesser skills of the surgeons and the lack of proper equipment in most Chinese hospitals. In order to reduce the gaps between hospitals of different levels, financial investments will have to be increased to improve the equipment available in low-level hospitals.

Both the 2013 ESCRS guidelines and the 2010 Chinese National Standard have recommended that broad-spectrum topical, intravitreal, and intravenous antibiotics (e.g., a combination of ceftazidime and vancomycin) be used to treat postoperative endophthalmitis before culture results are available^4^. However, we found that more than 40% of the cases did not follow these recommendations. In some patients, only one type of antibiotic (vancomycin or others) was used when a negative culture result was found, and in other cases, the doctors prescribed antibiotics based on their experience, which often did not cover the cultured bacteria. In our study, the rate of culture positivity was low, at only 44.2%. However, it is really dangerous to treat with vancomycin alone in the negative cases, because the gram negative bacteria might be missed. Overall, the final visual outcome of the endophthalmitis cases was generally poor: only 32.7% of the cases had a visual acuities of better than 20/40. Obviously, a nonstandard antibiotic regimen was one of key reasons.

A presenting visual acuity of 20/1,000 or better, proper antibiotic therapy, and phacoemulsification were found to be significantly associated with a good visual outcome. Here, those patients undergoing phacoemulsification had an increased likelihood of having final vision better than 20/70, which was in accordance with Al-Mezaine’s report^24^. One possible explanation for this finding may be that the initial visual acuity in phacoemulsification is often better than that in the ECCE cases, so the initial visual acuity decrease in the post-phacoemulsification endophthalmitis cases might be more significant than in the post-ECCE endophthalmitis cases. In addition, the presence of a large wound with sutures in the ECCE cases can cause a foreign body sensation that might mask the early symptoms of endophthalmitis. Therefore, the patients with post-phacoemulsification endophthalmitis may recognize the condition sooner and receive more timely treatment.

In summary, the occurrence rate of acute-onset endophthalmitis following cataract surgery was 0.11% in Chinese small and medium-scale departments of ophthalmology, which lags behind the levels of developed countries and Chinese top eye centers. Our findings suggest that intraoperative PCR with vitreous loss and a small hospital volume are major risk factors for developing endophthalmitis, while irrigation with a 0.5% PVP-I solution was an effective prophylaxis against it. Overall, phacoemulsification, better visual acuity at presentation, and proper antibiotic therapy are good predictors of a positive prognosis. Increasing financial investments and continuing education for physicians are expected to improve the level of care provided by most hospitals.

## Methods

The study design was a retrospective, consecutive case series. We reviewed patients who underwent standard phacoemulsification or extracapsular cataract extraction (ECCE) with intraocular lens (IOL) implantation in 30 small and medium-scale departments of ophthalmology, between January 1, 2011 and December 31, 2013. We collected all cases with acute-onset endophthalmitis who were diagnosed within 6 weeks of cataract surgery. The small and medium-scale departments of ophthalmology were defined as those with cataract surgery volumes ranging from 10 to 100 cases per month. We randomly sampled 30 departments from each region of China, which were distributed evenly, and located in cities with different economic levels.

Those patients who underwent combined procedures, such as penetrating keratoplasty or trabeculectomy, were not included in our study. In addition, those patients with presumed traumatic endophthalmitis, endogenous endophthalmitis, and delayed-onset endophthalmitis (6 weeks after surgery) were also excluded. This study was conducted under the approval of the Ethical Review Committees of these 30 hospitals, and conformed to the tenets of the Declaration of Helsinki^25^. Informed consent was obtained from all of the subjects.

The procedures were performed according to standardized techniques. In the phacoemulsification, a 3-mm superior scleral tunnel incision was made in 7.0%, a limbus tunnel incision was made in 18.8%, and a clear corneal incision was made in 74.2% of the patients. Then, a continuous curvilinear capsulorhexis was performed. After the emulsification of the lens, a foldable IOL was inserted, and the wound was hydrated. With the ECCE, a 9-mm superior scleral tunnel incision was made, a can-opener capsulotomy was performed, and a polymethyl methacrylate (PMMA) IOL was implanted. The wound was then closed with a minimum of 5 interrupted 10–0 nylon sutures.

For the perioperative infection prophylaxis, preoperative topical antibiotics were routinely administered for 1–3 days, while postoperative topical antibiotics (levofloxacin or tobramycin) and steroid eye drops were prescribed for 1–2 weeks. The other prophylaxis procedures differed among the 30 hospitals (Table 5). The patients were followed up postoperatively on the first day and at 1 week, 1 month, and 2 months afterwards.

Acute-onset endophthalmitis was clinically diagnosed according to a standardized protocol (ICD-9 code 360.0), and the details were explained in our previous study^4^. When a diagnosis of endophthalmitis was made, the patient immediately underwent an aqueous and/or vitreous tap and/or vitrectomy to isolate any organisms. Both culture-positive and culture-negative cases were included, and after sampling the organisms, antibiotic therapy began. A pars plana vitrectomy was applied to the patients who presented with light vision, light perception, or worse^26^,^27^.

The data collected from the patients included the following: age, gender, systemic disease, inpatient or outpatient status, hospital characteristics (surgery volume, geographic pattern, and general level), infection prophylaxis (e.g., 0.5% povidone iodine for the conjunctiva preparation or not), type of surgery (phacoemulsification or ECCE), location and size of incision, complications during surgery, time to onset of endophthalmitis, visual acuity at presentation, diagnostic procedures performed, culture results, treatment, and follow-up time.

The occurrence rate of acute-onset endophthalmitis following cataract surgery was calculated by each department, based on the first nine factors mentioned above. The 95% CIs for the occurrence rate estimates were calculated assuming a binomial proportion. A multivariate analysis was used to determine whether the factors were independent risk factors for the occurrence of endophthalmitis. A logistic regression model based on the 50 endophthalmitis patients (minus 2 cases with a lack of presenting visual acuity) was used to identify any possible factors associated with achieving a good visual outcome (a final BCVA of 20/70 or better). A p-value of less than 0.05 was considered to be statistically significant, and all of the analyses (except when noted) were performed using the Statistical Package for the Social Sciences (SPSS) version 22.0 (SPSS Inc., Chicago, Illinois, USA).

## Additional Information

**How to cite this article:** Zhu, Y. *et al*. The occurrence rate of acute-onset postoperative endophthalmitis after cataract surgery in Chinese small- and medium-scale departments of ophthalmology. *Sci. Rep.* **7**, 40776; doi: 10.1038/srep40776 (2017).

**Publisher's note:** Springer Nature remains neutral with regard to jurisdictional claims in published maps and institutional affiliations.

## Figures and Tables

**Table 1:** Occurrence rate of acute-onset postoperative endophthalmitis among different hospitals.

Hospital	Region	General level of located city	Endophthal-mitis Cases (n)	Effective cataract surgeries (n)	Total cataract surgeries (n)	Occurrence rate per 100 (95% Confidence Interval)
1	EC	3-tier	3	902	2050	0.33 (0.00, 0.71)
2	EC	4-tier	1	386	1021	0.26 (0.00, 0.77)
3	EC	4-tier	1	710	1040	0.14 (0.00, 0.42)
4	EC	4-tier	1	606	711	0.17 (0.00, 0.49)
5	EC	1-tier	2	1250	1610	0.16 (0.00, 0.38)
6	EC	1-tier	1	2641	2713	0.04 (0.00, 0.11)
7	EC	3-tier	3	1458	3033	0.21 (0.00, 0.44)
8	EC	2-tier	1	2159	2503	0.05 (0.00, 0.14)
9	EC	4-tier	1	761	1174	0.13 (0.00, 0.39)
10	EC	3-tier	1	2922	2932	0.03 (0.00, 0.10)
11	SC	3-tier	6	3360	3487	0.18 (0.04, 0.32)
12	SC	3-tier	1	586	1348	0.17 (0.00, 0.50)
13	SC	4-tier	1	3145	3598	0.03 (0.00, 0.09)
14	SC	3-tier	1	995	3195	0.10 (0.00, 0.30)
15	NC	1-tier	2	3180	3400	0.06 (0.00, 0.15)
16	NC	1-tier	1	1880	1976	0.05 (0.00, 0.16)
17	NC	3-tier	2	1526	2014	0.13 (0.00, 0.31)
18	NC	3-tier	1	2319	3012	0.04 (0.00, 0.13)
19	NC	1-tier	1	846	920	0.12 (0.00, 0.35)
20	NC	1-tier	1	638	668	0.16 (0.00, 0.46)
21	NC	4-tier	1	1648	2480	0.06 (0.00, 0.18)
22	NC	4-tier	4	666	912	0.60 (0.01, 1.19)
23	NC	2-tier	2	1234	1371	0.16 (0.00, 0.39)
24	MWC	3-tier	1	1442	1847	0.07 (0.00, 0.21)
25	MWC	2-tier	1	578	765	0.17 (0.00, 0.51)
26	MWC	4-tier	1	486	526	0.21 (0.00, 0.61)
27	MWC	3-tier	6	1857	2476	0.32 (0.06, 0.58)
28	MWC	4-tier	1	1957	2129	0.05 (0.00, 0.15)
29	MWC	3-tier	1	3209	3549	0.03 (0.00, 0.09)
30	MWC	4-tier	2	838	932	0.24 (0.00, 0.57)
Total	—	—	52	46185	59392	0.11 (0.08, 0.14)

EC, East China; SC, South China; NC, North China; MWC, Midwest China.

**Table 2:** Results of Bacterial Cultures in Patients With Endophthalmitis (n = 52).

Bacterial Cultures/Causative Organisms	Eye (n)	Origin of Specimen	
		aqueous humor (n)	Vitreous (n)
Negative bacterial cultures	29	8	21
Positive bacterial cultures	23	7	16
Gram-positive coagulase-negative	13	5	8
S. epidermidis	10	4	6
S. warneri	1	0	1
S. capitis	2	1	1
Other Gram-positive	7	2	5
S. aureus	2	0	2
E. faecalis	3	2	1
P. acnes	2	0	2
Gram-negative	3	0	3
P.aeruginosa	3	0	3

**Table 3:** Risk Factor for acute-onset postoperative Endophtalmitis after Cataract Surgery from a Multiple Logistic Regression Model.

Risk factor		category	No. of endophthalmitis cases	No. of Effective Cataract Surgeries	Occurrence rate per 100 (95% Confidence Interval)	Multivariate OR (95% Confidence Interval)	p-value
Age		<60	9	9624	0.094(0.086, 0.101)	Reference	0.889
		60–69	19	15122	0.126(0.119, 0.133)	1.34(0.61, 2.97)	
		70–79	17	15773	0.108(0.101, 0.114)	1.15(0.51, 2.59)	
		≥80	7	5666	0.124(0.112, 0.135)	1.32(0.49, 3.55)	
Gender		Male	20	24316	0.082(0.078, 0.087)	Reference	0.199
		Female	32	21869	0.146(0.140, 0.152)	1.44(0.82, 2.52)	
hospital	CSV/Y	<500	17	8335	0.204(0.193, 0.216)	2.21*(1.24, 3.94)	0.006
		≥500	35	37850	0.093(0.089, 0.096)	Reference	
	Geographic pattern	EC	9	8086	0.111(0.117, 0.134)	1.02(0.45, 2.34)	0.977
		SC	15	13795	0.109(0.102, 0.116)	Reference	
		NC	15	13937	0.108(0.101, 0.114)	0.99(0.48, 2.03)	
		MWC	13	10367	0.125(0.117, 0.134)	1.15(0.55, 2.43)	
	General level of located city	1-tier	8	10435	0.077(0.070, 0.083)	Reference	0.627
		2-tier	4	3971	0.101(0.088, 0.113)	1.31(0.40, 4.37)	
		3-tier	26	20576	0.126(0.120, 0.132)	1.65(0.75, 3.65)	
		4-tier	14	11203	0.125(0.117, 0.133)	1.63(0.68, 3.89)	
Type of surgery		Phaco	47	40848	0.115(0.111, 0.119)	0.81(0.32, 2.05)	0.662
		ECCE	5	5337	0.094(0.083, 0.104)	Reference	
Type of incision		Clear corneal	37	30310	0.122(0.117, 0.127)	Reference	0.718
				limber tunnel	7	7679		0.091(0.083, 0.100)	0.75(0.33, 1.68)
				scleral tunnel	8	8196		0.098(0.089, 0.106)	0.80(0.37,1.72)
Place of surgery		Inpatient	51	43326	0.118(0.114, 0.122)	Reference	0.201		
		Outpatient	1	2859	0.035(0.026, 0.044)	0.30(0.04, 2.15)			
0.5% PVP-I		absence	25	16123	0.155(0.148, 0.163)	1.73*(1.01, 2.98)	0.046		
		use	27	30062	0.090(0.086, 0.094)	Reference			
PCR		Yes	3	1625	0.185(0.160, 0.209)	1.68(0.52, 5.40)	0.614		
		No	49	44560	0.110(0.106, 0.114)	Reference			
PCR with vitreous loss		Yes	3	623	0.482(0.430, 0.533)	4.40*(1.34 14.14)	0.034		
		No	49	44560	0.110(0.106, 0.114)	Reference			
PCR without vitreous loss		Yes	0	1002	0.000 (0.000, 0.000)	—	0.550		
		No	49	44560	0.110(0.106, 0.114)				

PVP-I, povidone iodine; CSV/Y, cataract surgery volume/year; Phaco, phacoemulsification; PCR, posterior capsular rupture.

EC, East China; SC, South China; NC, North China; MWC, Midwest China.

*Statistically significant P value (<0.05).

**Table 4:** Logistic regression analysis of factors associated with achieving a final BCVA of 20/70 or better.

	Β Coefficient	p-value	Exp of Β	95% CI
Phacoemulsification	2.830	0.047	16.945	1.037, 276.832
Presenting visual acuity ≥20/1000	2.007	0.035	7.074	1.153, 43.413
Proper antibiotics	1.956	0.01	7.439	1.632, 33.907
Constant	−4.345			

Exp, exponentiation; CI, Confidence Interval.

**Table 5:** Infection prophylaxes of 30 hospitals.

Hospital	Lash trimming	0.5% PVP-I solution application	Antibiotics in irrigating solutions	Subconjunctival antibiotics	Ophthalmic ointment application
1	−	+	−	−	0.3% tobramycin
2	+	−	−	−	0.3% tobramycin
3	+	+	−	−	0.3% tobramycin
4	+	−	−	−	−
5	−	+	−	−	0.3% tobramycin
6	−	+	−	−	0.3% tobramycin
7	−	+	−	−	0.3% tobramycin
8	+	+	−	−	−
9	+	−	−	−	0.3% tobramycin
10	−	+	−	−	0.3% tobramycin
11	+	−	Tobramycin 16 mg/l	Tobramycin 4 mg/0.1 ml	0.3% tobramycin
12	−	−	Getamicin 8 mg/l	Tobramycin 4 mg/0.1 ml	0.3% tobramycin
13	−	+	−	−	0.3% tobramycin
14	−	+	−	−	0.3% tobramycin
15	−	−	−	−	0.3% tobramycin
16	+	+	−	Tobramycin 4 mg/0.1 ml	0.3% tobramycin
17	+	−	−	−	0.3% tobramycin
18	+	+	−	−	0.3% tobramycin
19	−	−	Getamicin 8 mg/l	−	−
20	−	+	−	Tobramycin 4 mg/0.1 ml	−
21	+	−	−	−	−
22	+	−	−	−	0.3% tobramycin
23	−	−	−	−	0.3% tobramycin
24	−	+	−	−	0.3% tobramycin
25	−	+	−	−	0.3% tobramycin
26	+	−	−	−	0.3% tobramycin
27	−	+	−	−	0.3% tobramycin
28	+	+	−	−	−
29	−	+	−	−	0.3% tobramycin
30	−	−	−	−	0.3% tobramycin

PVP-I, povidone-iodine.
